# The Risk of Severe Infections Following Rituximab Administration in Patients With Autoimmune Kidney Diseases: Austrian ABCDE Registry Analysis

**DOI:** 10.3389/fimmu.2021.760708

**Published:** 2021-10-29

**Authors:** Balazs Odler, Martin Windpessl, Marcell Krall, Maria Steiner, Regina Riedl, Carina Hebesberger, Martin Ursli, Emanuel Zitt, Karl Lhotta, Marlies Antlanger, Daniel Cejka, Philipp Gauckler, Martin Wiesholzer, Marcus Saemann, Alexander R. Rosenkranz, Kathrin Eller, Andreas Kronbichler

**Affiliations:** ^1^ Division of Nephrology, Department of Internal Medicine, Medical University of Graz, Graz, Austria; ^2^ Medical Faculty, Johannes Kepler University Linz, Linz, Austria; ^3^ Department of Internal Medicine IV, Section of Nephrology, Klinikum Wels-Grieskirchen, Wels, Austria; ^4^ Institute for Medical Informatics, Statistics and Documentation, Medical University of Graz, Graz, Austria; ^5^ Department of Internal Medicine I, University Hospital of St. Poelten, Karl Landsteiner University of Health Sciences, Karl Landsteiner Institute for Nephrology and Hematooncology, St. Poelten, Austria; ^6^ Department of Internal Medicine 3 (Nephrology and Dialysis), Feldkirch Academic Teaching Hospital, Feldkirch, Austria; ^7^ Department of Internal Medicine 2, Kepler University Hospital and Johannes Kepler University, Linz, Austria; ^8^ Department of Medicine III-Nephrology, Hypertension, Transplantation, Rheumatology, Geriatrics, Ordensklinikum Linz-Elisabethinen Hospital, Linz, Austria; ^9^ Department of Internal Medicine IV, Nephrology and Hypertension, Medical University Innsbruck, Innsbruck, Austria; ^10^ Department of 6Internal Medicine with Nephrology and Dialysis with Outpatient Department, Clinic Ottakring, Vienna, Austria; ^11^ Department of Medicine, University of Cambridge, Cambridge, United Kingdom

**Keywords:** rituximab, infections, glomerular disease, vasculitis, lupus, nephrotic, nephritic

## Abstract

**Objective:**

To characterize the incidence, type, and risk factors of severe infections (SI) in patients with autoimmune kidney diseases treated with rituximab (RTX).

**Methods:**

We conducted a multicenter retrospective cohort study of adult patients with immune-related kidney diseases treated with at least one course of RTX between 2015 and 2019. As a part of the ABCDE Registry, detailed data on RTX application and SI were collected. SI were defined by Common Terminology Criteria for Adverse Events v5.0 as infectious complications grade 3 and above. Patients were dichotomized between “nephrotic” and “nephritic” indications. The primary outcome was the incidence of SI within 12 months after the first RTX application.

**Results:**

A total of 144 patients were included. Twenty-five patients (17.4%) presented with SI, mostly within the first 3 months after RTX administration. Most patients in the nephritic group had ANCA-associated vasculitis, while membranous nephropathy was the leading entity in the nephrotic group. Respiratory infections were the leading SI (n= 10, 40%), followed by urinary tract (n=3, 12%) and gastrointestinal infections (n=2, 8%). On multivariable analysis, body mass index (BMI, 24.6 kg/m^2^ *versus* 26.9 kg/m^2^, HR: 0.88; 95%CI: 0.79-0.99; p=0.039) and baseline creatinine (HR: 1.25; 95%CI: 1.04-1.49; p=0.017) were significantly associated with SI. All patients in the nephritic group (n=19; 100%) who experienced a SI received oral glucocorticoid (GC) treatment at the time of infection. Hypogammaglobulinemia was frequent (58.5%) but not associated with SI.

**Conclusions:**

After RTX administration, impaired kidney function and lower BMI are independent risk factors for SI. Patients with nephritic glomerular diseases having concomitant GC treatment might be at higher risk of developing SI.

## Introduction

Severe infections (SI) are a major cause of morbidity and mortality in patients with kidney disease. In particular, glomerulopathies, either primary forms or secondary to systemic disorders, exhibit a heightened risk for such complications, which is determined by the underlying disease but to a major extent a direct consequence of immunosuppressive therapy.

Rituximab (RTX), a chimeric monoclonal antibody directed against the B cell CD20 antigen, was initially approved for the treatment of hematologic malignancies and subsequently for rheumatoid arthritis. In 2010, its label was expanded for the treatment of ANCA-associated vasculitis (AAV). In parallel, it has become an important off-label agent in the treatment of various forms of autoimmune kidney diseases, such as primary membranous nephropathy (MN), minimal change disease (MCD), and immune-mediated forms of focal segmental glomerulosclerosis (FSGS). While efficacy data of RTX in most glomerular pathologies are still limited to observational studies, several randomized controlled trials (RCT) investigating RTX in MN have recently been published (1–3).

Although the safety profile of RTX is considered favorable, SI can occur during and after anti-CD20 therapy. While few infectious complications were reported in recent key trials, a French study, involving 98 individuals with various types of glomerulopathies treated with RTX, noted infectious episodes in a quarter of patients; cumulative RTX dose and kidney failure were identified as independent risk factors (4). Generally, infectious complications appear to be mainly determined by the indication of RTX, i.e. the underlying disease, individual patient characteristics, and concurrent therapy.

In a large, contemporary multicenter cohort, we assessed incidence, type and risk factors for SI in patients treated with rituximab. Moreover, we wanted to investigate whether SI differ between “nephrotic “(e.g. MN, MCD, FSGS) and “nephritic” (AAV, lupus nephritis (LN)) indications. As the classification of autoimmune kidney disease is becoming increasingly granular and treatment approaches are being constantly refined, such data are important to allow for more individualized recommendations regarding prophylaxis strategies.

## Materials and Methods

### Patient Population and Data Collection

The Austrian B-Cell Depletion Evaluation (ABCDE) study is based on a multicenter retrospective data collection of adult patients with immune-related kidney diseases, treated with at least one course of RTX. Austrian tertiary centers for the management of autoimmune kidney diseases were invited to participate in this National Registry. From January 2015 until December 2019 data on 144 patients from the participating centers were collected.

The registry contains data from patients with any of the following disorders: AAV, FSGS, immunoglobulin G4-related disease (IgG4RD), LN, MCD, MN and membranoproliferative glomerulonephritis (MPGN). Additional inclusion criteria consisted of age ≥ 18 years at the start of RTX therapy and a minimum follow-up time of 3 months after the first RTX application.

Data on clinical characteristics, comorbidities, prior immunosuppressives [i.e., calcineurin inhibitors (CNI), cyclophosphamide (CYC), and mycophenolate mofetil (MMF)], and baseline laboratory data of all patients were collected. Additionally, detailed data on RTX application including dose, treatment line, concomitant glucocorticoid treatment, and further maintenance therapies, were gathered. Patients were treated with RTX in accordance with their physician’s standard practice. Dialysis was defined as newly started kidney replacement therapy (KRT) after first RTX administration.

Serial measurements on immunoglobulin G (IgG) and absolute neutrophil count (ANC) were conducted during the observation period. Hypogammaglobulinemia and neutropenia were classified by nadir IgG levels (IgG levels < 7 g/L) (5) and ANC (neutrophil count <1.5 x 10^9^/L) (defined by the local laboratory) during the observation period. In addition, data on occurrence of malignancies (solid tumors and malignant hematological disorders) during RTX therapy were also recorded.

All data, if available, were derived retrospectively from the electronic medical records of the attending centers. The date of the first RTX application (index date) was registered to calculate the time to outcome event. The duration of RTX therapy was measured from the time of drug initiation to discontinuation of the drug or censored at the date of the last follow-up for patients remaining on the drug at the time of data analysis. Data were collected until patient death, loss of follow-up, or end of the follow-up on 31 December 2019.

### Definition of Infections and Outcomes

The NCI Common Terminology Criteria for Adverse Events Version 5.0 (NCI-CTCAE v5.0) was used to define and grade infections. Clinical and hospitalization reports were reviewed during RTX treatment to identify severe (grade 3-5) infections. Notably, mild infections (grade 1-2) were not considered. The site of SI was defined as respiratory, gastrointestinal, urinary tract, and other infections. Additionally, the daily corticosteroid dose (prednisolone equivalent) at the time of infection was collected.

The primary outcome of this study was the incidence of SI within 12 months after the first RTX application. In addition, incidence rates of hypogammaglobulinemia and neutropenia, as well as new onset of malignancies, were also calculated.

### Ethical Statement

The study protocol was approved by the institutional ethics committee of the Medical Faculty of the Johannes Kepler University, Linz, Austria (Nr. 1117/2018) and subsequently by the local ethics committee of all participating centers. Informed consent was not obtained from the participants, as it was a noninterventional retrospective data analysis of real-life data collected on patients’ regular visits. The study was conducted under the principles of the Declaration of Helsinki.

### Statistical Analysis

Continuous parameters are summarized as the median and range (minimum, maximum) and categorical parameters are presented as absolute and relative frequencies. Baseline characteristics and investigated possible risk factors for SI at start of RTX therapy included age, BMI, sex, creatinine level, comorbidities, prior IS and RTX induction protocol are presented for all patients, and for nephrotic (including MN, MCD, FSGS) and nephritic (including AAV, LN, MPGN, IgG4RD) patients separately. Missing specific data were not imputed. Characteristics between both groups are compared by Mann–Whitney U and Fisher’s exact test. To compare incidences of SI between the groups and accounting for the different baseline characteristics, inverse probability treatment weighting (IPTW) was performed. The propensity score was calculated by using logistic regression with group as outcome and including age, BMI, sex, creatinine level, comorbidities (yes/no) and prior IS (yes/no) as covariates. Additionally, Kaplan Meier curves for 12-month SI are presented for nephrotic and nephritic patients. To evaluate risk factors for SI within 12 months after RTX therapy start, univariable Cox proportional hazard regression analyses were performed. Time to event is defined as time from start of RTX to SI or death, lost to follow up or month 12, whatever occurs first. In a multivariable Cox regression model, all parameters with a *p*-value < 0.2 were included. Results are presented as Hazard ratios (HR) and their corresponding 95% confidence intervals (CIs). A *p*-value < 0.05 was considered statistically significant, and all analyses were performed using SAS version 9.4 (SAS Institute, Cary, NC, USA).

## Results

### Patient Characteristics

One hundred forty-four patients with autoimmune kidney diseases were included. Detailed baseline demographic and clinical characteristics are shown in **Table 1**. Eighty-three patients had a nephritic glomerular disease, while 61 patients belonged to the nephrotic group. The majority of patients had AAV in the nephritic, while MN was the leading diagnosis in the nephrotic group (54% and 33% of all patients, respectively). The comorbidities were comparable in both groups. A significantly higher baseline median creatinine was observed in the nephritic group as compared to the nephrotic patients (1.7 [range: 0.7-15.4] *vs*. 1.2 [range: 0.6-2.8] mg/dL, p<0.001) at the time of RTX initiation. Nearly 40% of the patients in both groups received an induction therapy protocol using 2 x 1000 milligram (mg) RTX a fortnight apart or 4 x 375 mg/m^2^ in weekly intervals, respectively, while fifty percent of all patients received RTX as maintenance therapy.

**Table 1:** Baseline patient characteristics in the whole study population and stratified according to the classification on nephritic or nephrotic groups.

	Whole study population N = 144	Patients with nephritic diseases N = 83	Patients with nephrotic diseases N = 61	p-value
Age (years)	61.2 (20.4, 83.8)	65.1 (21.1, 83.8)	57.6 (20.4, 78.7)	**0.012**
BMI (kg/m^2^)	26.2 (17.7, 39.9)	25.6 (17.7, 37.6)	27.3 (19.9, 39.9)	**0.036**
Female sex, n (%)	51 (35.4)	39 (47.0)	12 (19.7)	**<0.001**
Diagnosis, n (%)				
AAV	78 (54.2)	78 (94.0)	–	–
LN	3 (2.1)	3 (3.6)	–	–
MPGN	1 (0.7)	1 (1.2)	–	–
IgG4RD	1 (0.7)	1 (1.2)	–	–
MN	48 (33.3)	–	48 (78.7)	–
MCD	8 (5.6)	–	8 (13.1)	–
FSGS	5 (3.5)	–	5 (8.2)	–
Comorbidities, n (%)				
Pulmonary disease	12 (8.3)	7 (8.4)	5 (8.2)	1
Cardiovascular disease	30 (20.8)	15 (18.1)	15 (24.6)	0.408
Diabetes mellitus	17 (11.8)	9 (10.8)	8 (13.1)	0.795
Arterial hypertension	93 (64.6)	53 (63.9)	40 (65.6)	0.862
Dialysis (any time), n (%)	25 (17.4)	25 (30.1)	0 (0)	**<0.001**
Creatinine (mg/dL)*	1.3 (0.6, 15.4)	1.7 (0.7, 15.4)	1.2 (0.6, 2.8)	**<0.001**
Prior IS, n (%)				
MMF	17 (11.8)	11 (13.3)	6 (9.8)	0.608
CNI	29 (20.1)	0 (0)	29 (47.5)	**<0.001**
CYC	53 (36.8)	49 (59.0)	4 (6.6)	**<0.001**
RTX induction protocol, n (%)				
1000 mg (2x/2-weeks apart)	62 (43.1)	34 (41.0)	28 (45.9)	**0.001**
375 mg/m^2^ (4x/weekly)	64 (44.4)	32 (38.6)	32 (52.5)	
Other	18 (12.5)	17 (20.5)	1 (1.6)	
RTX maintenance, n (%)	72 (50.0)	49 (59.0)	23 (37.7)	**0.018**

Statistically significant p-values appear in boldface type (p < 0.05). Continuous variables are expressed as median (minimum and maximum). Categorical variables are n (%). *Non-dialysis dependent patients. AAV, anti-neutrophil cytoplasmatic antibody (ANCA), associated vasculitis; BMI, body mass index; CNI, calcineurin inhibitor; CYC, cyclophosphamide; FSGS, focal segmental glomerulosclerosis; GN, glomerulonephritis; IgG4RD, immunoglobulin G4-related disease; IS, immunosuppression; LN, lupus nephritis; MCD, minimal change disease; MN, membranous nephropathy; MMF, mycophenolate-mofetil; MPGN, membranoproliferative glomerulonephritis; RTX, rituximab.

### Incidence Rate and Type of Severe Infections

**Figure 1:**
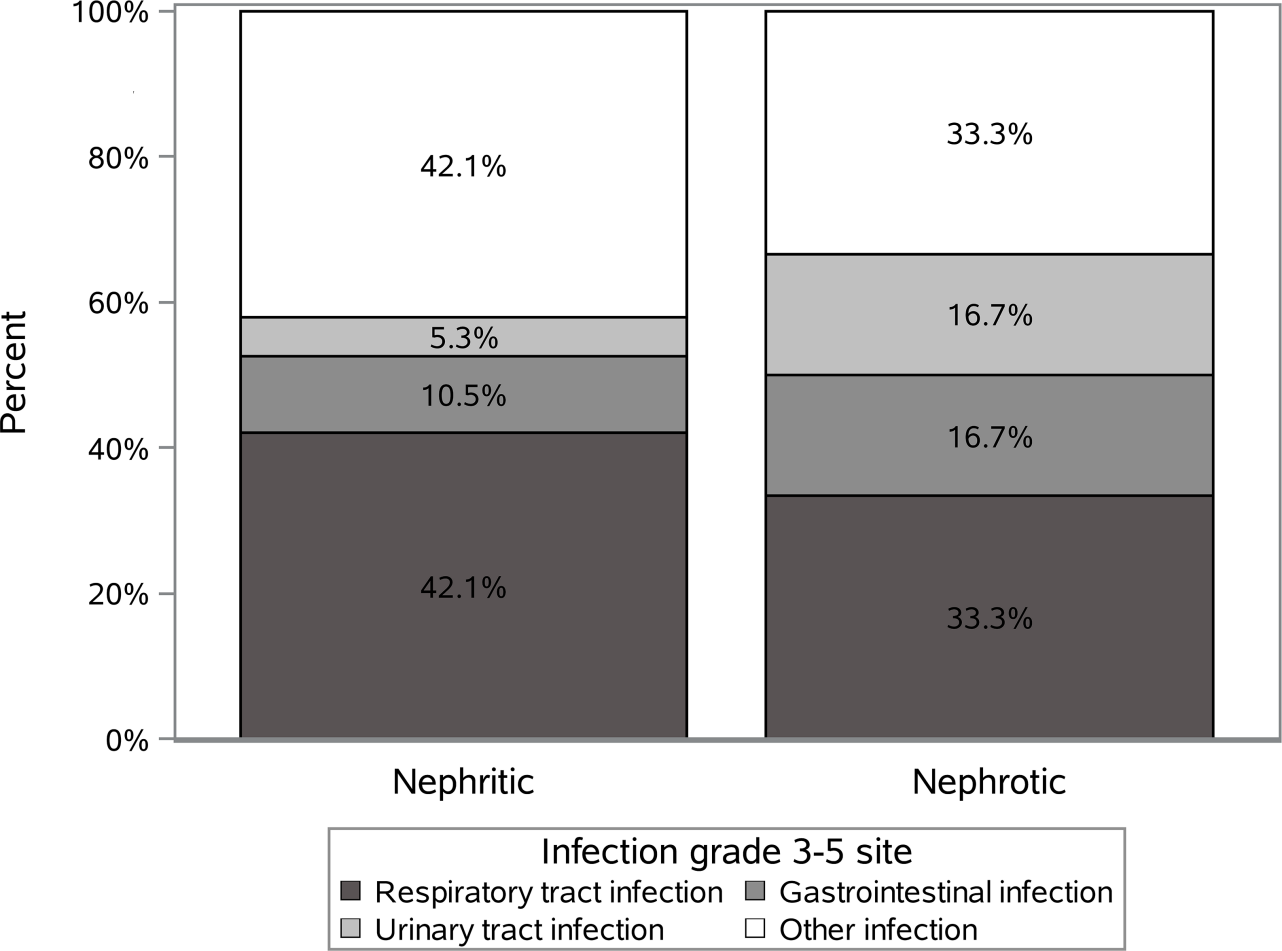
The distribution of all severe infections according to the infection sites in the nephritic and nephrotic groups.

Twenty-five out of 144 patients (17.4%) presented with SI during a median follow-up time of 2.2 (0-4.9) years. Respiratory infections were the most frequent ones, but the majority of patients (58% and 67% nephritic and nephrotic groups) had other sites affected. The distribution of all SI according to the infection sites is shown in **Figure 1**. Two patients had a SI of grade 4 and 5 (n=1, respectively, both in nephritic group) according to the CTCAE v5.0 criteria.

In the long term, patients in the nephritic group tended to have more SI compared to nephrotic group patients (n=19, 22.9% *vs*. n=6, 10.2%; HR=1.82, 95% CI: 0.73-4.54, p=0.198). The median time to the first SI was 139 (range: 17-1345) days. Patients in the nephrotic group had a clearly shorter time until experiencing their first infection as compared to those in the nephritic group (median: 35 [range: 17-274] *vs*. 259 [range: 20-1345] days, respectively; p=0.024).

**Figure 2:**
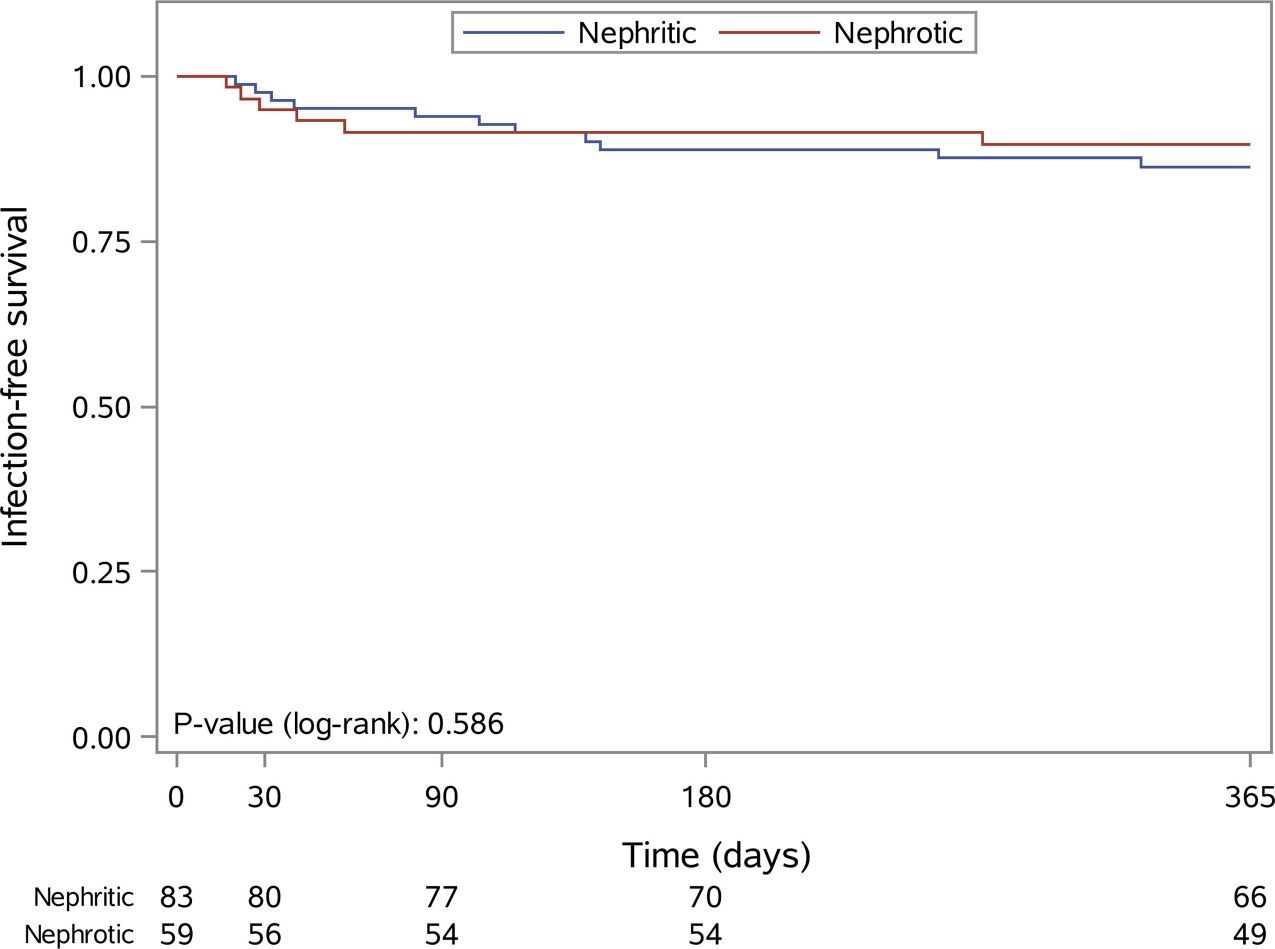
Kaplan-Meier curves for infection-free survival within the first 12 months after the first rituximab administration in patients with nephritic (blue line) and nephrotic (red line) syndrome.

Within 3 and 12 months after first RTX administration n=10 (7.0%) and n=17 (12.0%) SI were observed. For nephritic and nephrotic patients, the infection rates were 6% *versus* 8.5% within 3 months (HR=0.39, 95% CI: 0.10-1.51, p=0.172) and 13.3% *versus* 10.2% within 12 months (HR=0.91, 95% CI: 0.33-2.51. p=0.852). Kaplan Meier estimates for 12-month SI rates are presented in **Figure 2**.

### Predictors of Severe Infections

Univariable Cox regression analysis assessed predictors of SI within 12 months after first RTX administration (**Supplementary Table 1**). In multivariable analysis, BMI (hazard ratio [HR]: 0.88; 95% confidence interval [CI]: 0.79-0.99; p=0.039) and baseline creatinine (HR: 1.25; 95% CI: 1.04-1.49; p=0.017) significantly affected SI within 12 months after the first RTX administration (**Table 2**).

**Table 2:** Multivariable Cox regression analysis on the predictors of severe infections within 12 months after the first rituximab administration.

Covariate	Hazard ratio	95% confidence interval	p-value
**BMI (kg/m^2^)**	**0.88**	**0.79-0.99**	**0.039**
**Creatinine (mg/dL)**	**1.25**	**1.05-1.49**	**0.017**
Dialysis	0.90	0.22-3.76	0.887
Arterial hypertension	2.40	0.65 – 8.86	0.189

Statistically significant p-values appear in boldface type (p < 0.05). BMI, body mass index.

All the patients in the nephritic group (n=19; 100%) who experienced a SI after the first RTX administration received oral corticosteroid (CS) treatment at the time of the infection. The median dose of CS used at the time of infection was 7.5 (range: 2.5-50.0) mg. On the other hand, only two out of six (33%) patients were treated with oral CS (median dose: 26.3 [range: 15.0-37.5] mg) at the time of SI in the nephrotic group.

### Incidence Rate of Hypogammaglobulinemia, Neutropenia, and Malignancies

Hypogammaglobulinemia was observed in 72 out of 123 (58.5%) patients with serum IgG measurements. Most of these periods (40 out 72 [55.5%]) occurred within 12 months after the first RTX administration. Nineteen patients had a SI and hypogammaglobulinemia; however, no association between hypogammaglobulinemia and SI was observed (p=0.067). Interestingly, most of the infections (n=17) occurred before hypogammaglobulinemia was observed.

In total, 16 out of 140 (11.4%) patients with measurements experienced neutropenia, and it was significantly associated with SI during the whole observation period (p=0.030). Specifically, nine episodes of neutropenia (n=5 and n=4 in the nephritic and nephrotic groups, respectively) occurred during the first 12 months after the first RTX administration, which showed, however, no association with SI neither in the nephritic nor in the nephrotic group (p>0.05). Notably, no neutropenia was observed at the time of the first RTX administration, while only two patients were neutropenic at the time of the infection.

In addition, five patients (3.4%) experienced malignancy during the observation period. Two patients had a skin tumor, while one prostatic cancer, one breast cancer, and one malignant testicular tumor were observed in the other three patients.

## Discussion

The ABCDE Registry, an Austrian registry focusing on the use of RTX in kidney disease indications, included 144 patients, with the majority of patients having a diagnosis of AAV (54.2%) and MN (33.3%). During the observational period, 25 patients presented with a SI defined as CTCAE grade 3 and higher. Among these, there was no significant difference between nephritic and nephrotic glomerular diseases (22.9% *versus* 10.2%). Most infections occurred within the first 3 months of RTX use, and this was most prominent for nephrotic diseases (5 infections out of 6 in total).

This study found that for nephritic glomerular diseases the risk of SI might be high when glucocorticoids are concomitantly prescribed. All SI were recorded on background glucocorticoid use. Reduced glucocorticoid regimens showed comparable efficacy estimates in AAV (6, 7). In the PEXIVAS trial, the reduced-dose glucocorticoid regimen reached the primary end point, a combination of end-stage kidney disease and death from any cause, less frequently at one year of follow-up, although the differences were not significant. However, a significant reduction in SI at one year was observed with a reduction by over 30% in comparison to a standard-dose glucocorticoid regimen (6). More informative data on the use of a low-dose glucocorticoid regimen in combination with RTX was recently provided by the LOVAS trial, randomizing patients with AAV to either a reduced-dose or high-dose glucocorticoid regimen. The cumulative glucocorticoid dose was 1.3 g in the reduced-dose in comparison to 4.2 g in the high-dose arm, corresponding to a reduction of 68%. A similar proportion of patients achieved remission, while serious adverse events were reduced in the reduced-dose arm. Notably, only 7 SI in 5 patients were recorded in the reduced-dose arm in comparison to 20 events in 13 patients in the high-dose glucocorticoid arm (7). In the ABCDE Registry, fourteen of the 19 recorded SI in the nephritic group occurred beyond 3 months of follow-up, and 8 after 12 months. Studies such as LOVAS or the RAVE trial with a prescribed glucocorticoid withdrawal before month 6 after induction therapy highlighted both the safety and efficacy of such an approach (7, 8). A retrospective study from Germany subdivided patients by the use of a glucocorticoid dose of either below 7.5 mg or greater/equal to 7.5 mg by month 6. Patients with a higher glucocorticoid dose had more infectious episodes (1.7 *versus* 0.6), while the glucocorticoid dose had no impact on patient survival, kidney function, or relapse rate (9). The presented data of the ABCDE Registry also support a paradigm change towards a reduced time of glucocorticoid use. Notably, only 2 patients in the nephrotic group had concomitant glucocorticoid therapy, which argues against a significant impact of glucocorticoids on SI risk in this population.

Lower BMI (24.6 kg/m^2^ *versus* 26.9 kg/m^2^) was associated with higher risk of SI. A recent study from Japan involving 93 patients with a diagnosis of microscopic polyangiitis (MPA) subdivided patients into three groups, one group with low BMI (<18.5 kg/m^2^, n=22), one group with normal BMI (18.5-23.0 kg/m^2^, n=53) and one with high BMI (>23 kg/m^2^, n=18). SI were recorded in 63.6%, 24.5% and 11.1% of patients in the respective groups. Patients in the low and normal BMI group were more likely to suffer from a body weight loss > 10% within six months before diagnosis of MPA was made. Patients in the low and normal BMI group also exhibited higher mortality rates in comparison to patients in the high BMI group (10). Sub-analysis of the RAVE trial indicated that newly diagnosed patients have an increase in total and low-density lipoprotein cholesterol after achieving remission. Reduced lipid levels at baseline correlated with erythrocyte sedimentation rate (11), indicating that inflammatory processes may play a critical role explaining the initial weight loss and altered lipid levels observed in patients with AAV. This persistent inflammatory process may in part explain the higher risk of SI observed in these patients. Lower BMI might also indicate a higher disease severity and these patients might have received higher glucocorticoid doses at baseline, again explaining the higher risk of SI. Despite non-significant, patients with nephrotic syndrome had a numerically lower frequency of SI, and water retention/edema development leading to a higher BMI might also influence our findings.

Most SI in our analysis occurred within the first months of RTX administration, which is in line with several investigations. A single-center study including 221 patients with autoimmune indications found SI in 42 patients. The prevalence of infectious complications was most pronounced within the first three months of follow-up (7.2%) and increased to 15.5% at one and 17.8% at two years. Most patients presented with pneumonia (45%) and/or bacteremia (21%) (12). RTX use for several indications was associated with an increase in SI from 17.2% pre-rituximab to 21.7% after administration among 8633 patients. Again, most infections occurred within the first six months of RTX administration. Pre-existing hypogammaglobulinemia was a strong risk factor for SI before RTX initiation and a third of these patients experienced SI following RTX (13). Hypogammaglobulinemia was frequently observed in our cohort (72 out of 123 with measurements, 58.5%), but was not associated with SI in our study. Notably, the sample size might have impacted this finding. Another complication of RTX is late-onset neutropenia (14). Neutropenia was associated with SI in our analysis of the ABCDE Registry, as 8 out of 16 patients with neutropenia had a SI during follow-up. A large single-center study found at least one episode of late-onset neutropenia in 71/738 adult patients receiving RTX. Its occurrence is more frequent within the first year of RTX administration and is not observed in patients with minimal change disease or focal segmental glomerulosclerosis. A majority of patients was asymptomatic during the neutropenic episode, while 31.3% and 8.5% presented with fever and septicemia (15).

Further analyses revealed an association of baseline creatinine with the risk of SI, which remained significant after adjustment for dialysis-dependency. Similar results were reported from a French study focusing on a combined end point of infection and/or death. Twenty-six out of 98 patients reached the end point during follow-up and baseline creatinine showed a borderline significance (4). Baseline creatinine was comparable to our cohort. Most analyses focusing on patients with AAV found a similar tendency towards more SI in patients with impaired kidney function (16–20), which is in line with our results.

Infections are one of the leading complications of nephrotic syndrome. Our analysis indicated that patients with nephrotic syndrome are prone to infections particularly within the first three months after RTX administration. In MN, there is an increased risk to develop invasive pneumococcal infection and there is a recommendation that patients receive pneumococcal and annual influenza vaccination (21). Vaccination might mitigate the risk to develop severe courses of these infections, while the humoral response to vaccines is severely impaired by rituximab within the first six months (22) Antibiotic prophylaxis with trimethoprim/sulfamethoxazole, known to reduce SI in patients with AAV (17, 23), might be used in this vulnerable phase.

This study has several limitations. The ABCDE Registry was initiated as a retrospective survey among nephrology departments to capture complications of RTX therapy in glomerular diseases, before initiating a prospective study phase. The analyzed groups (nephritic and nephrotic) differed significantly in terms of age, BMI, sex, the history of immunosuppressive drug use and maintenance RTX administration, some of these factors potentially influencing our results. The presented study assessed data retrospectively, so limitations inherent with its retrospective character need to be considered (i.e. missing data, such as information on IgG levels). Initial data collection did not include the assessment of antibiotic prophylaxis used in these patients (i.e. *Pneumocystis jirovecii* prophylaxis). This information would be of particular interest in patients with nephrotic syndrome presenting with SI early after RTX use. Information about cumulative glucocorticoid use, the use of intravenous methylprednisolone to control initial disease activity and longitudinal follow-up of BMI values is missing, all of particular importance to understand the finding of a higher rate of SI in patients with lower BMI. The sample size of our study is small, but comparable to other investigations published, and multi-national efforts are needed to inform about frequency, severity and risk factors of SI in patients with glomerular diseases receiving rituximab.

In conclusion, analysis of the ABCDE Registry retained impaired kidney function and lower BMI as independent risk factors to develop SI after RTX administration. All infectious complications in patients with nephritic glomerular diseases occurred during concomitant GC treatment, while the risk might be independent of glucocorticoid use in nephrotic patients. Further studies are needed assessing the exact use of antibiotic prophylaxis, the capacity of antibiotics to mitigate the risk of SI, and the role of low-dose glucocorticoid protocols and influence on SI.

## Data Availability Statement

The original contributions presented in the study are included in the article/**Supplementary Material**. Further inquiries can be directed to the corresponding author.

## Ethics Statement

The studies involving human participants were reviewed and approved by Johannes Kepler University, Linz, Austria (Nr. 1117/2018). Written informed consent for participation was not required for this study in accordance with the national legislation and the institutional requirements.

## Author Contributions

MWin and AK initially designed the ABCDE Registry. BO, MWin, MK, MSt, CH, MU, EZ, KL, MA, DC, PG, MWie, MSa, ARR, KE, and AK contributed to data acquisition, drafting the manuscript and approving the manuscript. RR performed statistical analysis. The first draft of the manuscript was written by BO, MWin, RR and AK. All authors contributed to the article and approved the submitted version.

## Funding

The presented work received no funding.

## Conflict of Interest

The authors declare that the research was conducted in the absence of any commercial or financial relationships that could be construed as a potential conflict of interest.

## Publisher’s Note

All claims expressed in this article are solely those of the authors and do not necessarily represent those of their affiliated organizations, or those of the publisher, the editors and the reviewers. Any product that may be evaluated in this article, or claim that may be made by its manufacturer, is not guaranteed or endorsed by the publisher.
