# Supplementary material for: The Risk of Severe Infections Following Rituximab Administration in Patients With Autoimmune Kidney Diseases: Austrian ABCDE Registry Analysis
Source: Front Immunol. 2021 Oct 29;12:760708. doi: 10.3389/fimmu.2021.760708 (PMC8586204; doi:10.3389/fimmu.2021.760708)
Supplement: Supplementary file 1 [file Table_1.docx]

Supplementary Material

# Supplementary Tables

**Supplementary Table 1.** Univariable Cox regression analysis on the predictors of severe infections within 12 months after the first rituximab administration.

| **Covariate** | **No infection** | **Infection** | **Hazard**  **ratio** | **95% confidence**  **interval** | **p-value** |
| --- | --- | --- | --- | --- | --- |
| Age (years) | 61.2 (21.1, 83.8) | 65.1 (20.4, 81.7) | 1.001 | (0.968, 1.034) | 0.969 |
| **BMI (kg/m^2^)** | **26.9 (17.7, 38.1)** | **24.6 (19.5, 28.7)** | **0.882** | **(0.782, 0.995)** | **0.041** |
| **Creatinine (mg/dL)** | **1.3 (0.6, 12.0)** | **2.0 (1.1, 15.4)** | **1.283** | **(1.133, 1.452)** | **<0.001** |
| Nephritic syndrome | 72 (86.7%) | 11 (13.3%) | 1.318 | (0.487, 3.563) | 0.587 |
| Female sex | 46 (90.2%) | 5 (9.8%) | 0.749 | (0.264, 2.126) | 0.587 |
| Comorbidities |  | | | | |
| Pulmonary disease | 9 (81.8%) | 2 (18.2%) | 1.591 | (0.364, 6.962) | 0.537 |
| Cardiovascular disease | 26 (86.7%) | 4 (13.3%) | 1.188 | (0.387, 3.644) | 0.763 |
| Diabetes mellitus | 14 (82.4%) | 3 (17.6%) | 1.611 | (0.463, 5.608) | 0.453 |
| Arterial hypertension | 77 (84.6%) | 14 (15.4%) | 2.819 | (0.810, 9.811) | 0.103 |
| **Dialysis** | **19 (76.0%)** | **6 (24.0%)** | **2.769** | **(1.023, 7.493)** | **0.045** |
| Prior IS |  | | | | |
| MMF | 15 (93.8%) | 1 (6.3%) | 0.471 | (0.062, 3.553) | 0.465 |
| CNI | 23 (85.2%) | 4 (14.8%) | 1.35 | (0.440, 4.141) | 0.600 |
| CYC | 46 (86.8%) | 7 (13.2%) | 1.157 | (0.441, 3.041) | 0.767 |
| RTX induction protocol  (1000 mg as reference) |  | | | | |
| 1000 mg  (2x/2 weeks apart) | 52 (83.9) | 10 (16.1) | - | - | - |
| 375 mg  (4x/weekly) | 56 (90.3) | 6 (9.7) | 0.589 | (0.214, 1.622) | 0.306 |
| Other | 56 (90.3) | 6 (9.7) | 0.58 | (0.211, 1.596) | 0.291 |

Statistically significant *p*-values appear in boldface type (*p* < 0.05). Continuous variables are expressed as median (minimum, maximum). Categorical variables are n (%). Abbreviations: BMI: body mass index, CNI: calcineurin inhibitor, CYC: cyclophosphamide, IS: immunosuppression, MMF: mycophenolate-mofetil, RTX: rituximab
